# (*Z*)-Methyl 2-methoxy­imino-3-oxo­butanoate

**DOI:** 10.1107/S1600536808004376

**Published:** 2008-02-15

**Authors:** Jin-Yong Lu, Wei-Zheng Shen, Hans Preut, Hans-Dieter Arndt

**Affiliations:** a Fakultät Chemie, Technische Universität Dortmund, Otto-Hahn-Strasse 6, 44221 Dortmund, Germany; b Max-Planck-Institut für Molekulare Physiologie, Otto-Hahn-Strasse 11, 44221 Dortmund, Germany

## Abstract

The title compound, C_6_H_9_NO_4_, was prepared stereoselectively as a precursor for 1-aza­dienes in a study of hetero-Diels–Alder reactions. The configuration of the C=N double bond was found to be *Z*, corroborating earlier assignments of similar compounds based only on NMR and IR spectroscopic analysis.

## Related literature

For related literature, see: Buehler (1967 ▶); Corrêa & Moran (1999 ▶); Fletcher *et al.* (2006 ▶); François *et al.* (2004 ▶); Jirman *et al.* (1990 ▶); Karabatsos & Taller (1968 ▶); Levy & Nelson (1972 ▶); Lu & Arndt (2007 ▶).
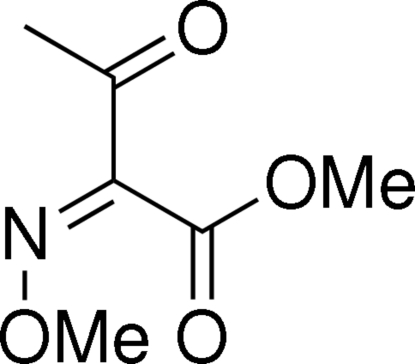


## Experimental

### 

#### Crystal data

C_6_H_9_NO_4_
*M* *_r_* = 159.14
Orthorhombic, 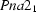

*a* = 8.3410 (17) Å
*b* = 13.410 (3) Å
*c* = 7.2900 (15) Å
*V* = 815.4 (3) Å^3^
*Z* = 4
Mo *K*α radiation
μ = 0.11 mm^−1^
*T* = 291 (1) K
0.2 × 0.2 × 0.2 mm

#### Data collection

Nonius KappaCCD diffractometer
Absorption correction: none
3104 measured reflections
899 independent reflections
536 reflections with *I* > 2σ(*I*)
*R*_int_ = 0.045

#### Refinement

*R*[*F* ^2^ > 2σ(*F* ^2^)] = 0.027
*wR*(*F* ^2^) = 0.057
*S* = 1.09
899 reflections
104 parameters
1 restraint
H-atom parameters constrained
Δρ_max_ = 0.08 e Å^−3^
Δρ_min_ = −0.11 e Å^−3^

### 

Data collection: *COLLECT* (Nonius, 1998 ▶); cell refinement: *DENZO* and *SCALEPACK* (Otwinowski & Minor, 1997 ▶); data reduction: *DENZO* and *SCALEPACK*; program(s) used to solve structure: *SHELXS97* (Sheldrick, 2008 ▶); program(s) used to refine structure: *SHELXL97* (Sheldrick, 2008 ▶); molecular graphics: *SHELXTL-Plus* (Sheldrick, 2008 ▶); software used to prepare material for publication: *SHELXL97* and *PLATON* (Spek, 2003 ▶).

## Supplementary Material

Crystal structure: contains datablocks I, global. DOI: 10.1107/S1600536808004376/hb2698sup1.cif

Structure factors: contains datablocks I. DOI: 10.1107/S1600536808004376/hb2698Isup2.hkl

Additional supplementary materials:  crystallographic information; 3D view; checkCIF report

## Figures and Tables

**Fig. 1.**
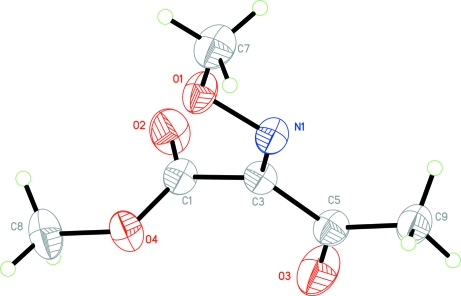
The molecular structure of (I) with displacement ellipsoids shown at the 30% probability level (arbitrary spheres for the H atoms).

## supplementary crystallographic information

### Comment

Oxime geometry has been found to be important for determining their reactivity in cycloadditions and pericyclic reactions (*e.g.* François *et al.*, 2004). The title compound, (I), was prepared in the study of hetero-Diels–Alder reactions to form 3-hydroxy-pyridines (Lu & Arndt, 2007; Fletcher *et al.*, 2006).

The crystal structure of (I) (Fig. 1) verifies earlier studies by NMR and IR (Buehler, 1967; Karabatsos & Taller, 1968; Levy & Nelson, 1972; Jirman *et al.*, 1990; Corrêa & Moran, 1999) of *Z*-configured oximes and forms a basis for further studies in the field. Interestingly, the C1/O2/O4 carboxyl group in (I) adopts a dihedral angle of 93° with respect to the coplanar N?C—C?O π-system, which indicates complete absence of electronic conjugation.

### Experimental

A stirred solution of 7.25 g (50.0 mmol) of *Z*-Methyl 2-(hydroxyimino)-3-oxobutanoate (Lu & Arndt, 2007; Fletcher *et al.*, 2006) in anhydrous acetone (50 ml) was cooled to 273 K and potassium carbonate (3.8 g, 27.5 mmol) was added, followed by dimethyl sulfate (5.70 ml, 60.0 mmol). The stirred reaction mixture was warmed to room temperature over 2 h and kept stirring for 10 h (TLC control). The reaction mixture was filtered and the solid residue was rinsed with acetone (3 × 10 ml). The combined filtrates were evaporated to dryness, redissolved in Et_2_O (100 ml), washed with sat. NaCl solution (3 × 40 ml) and dried with Na_2_SO_4_. Concentration and purification by column chromatography (100 g SiO_2_, EtOAc/light petroleum *v*/*v* = 1:8) gave 7.60 g (47.8 mmol, 96%) of the title compound as a colourless oil which crystallized on standing as colourless cubes.

Mp = 335–337 K; *R*_f_ = 0.46 (SiO_2_, EtOAc/cyclohexane = 1:2); ^1^H NMR (400 MHz, CDCl_3_) δ = 2.38 (3*H*, s, C(O)CH3), 3.85 (3*H*, s, =NOCH_3_), 4.08 (3*H*, s, COOCH_3_); ^13^C NMR (100.6 MHz, CDCl_3_) δ = 25.1 (C(O)CH_3_), 52.5 (COOCH_3_), 64.4 (NOCH_3_), 149.9 (C=N), 161.5 (COOCH_3_), 192.7 (C(O)CH_3_); IR (KBr): ν = 3009w, 2951w, 1744 s, 1683 s, 1596 s, 1241 s, 1021 s, 841 s cm^-1^; HRMS (EI): m/*Z* calc. for C_6_H_9_NO_4_ [*M*^+^]: 159.0532, found: 159.0524.

### Refinement

Anomalous dispersion was negligible and Friedel pairs were merged before refinement.

The H atoms were placed in calculated positions, with C—H = 0.96 Å and were refined as riding, with *U*_iso_(H) = 1.5*U*_eq_(C); the methyl groups were allowed to rotate but not to tip.

### Crystal data

**Table:**

C_6_H_9_NO_4_	*F*_000_ = 336
*M**_r_* = 159.14	*D*_x_ = 1.296 Mg m^−^^3^
Orthorhombic, *P**n**a*2_1_	Mo *K*α radiation λ = 0.71073 Å
Hall symbol: P 2c -2n	Cell parameters from 3104 reflections
*a* = 8.3410 (17) Å	θ = 3.0–27.5º
*b* = 13.410 (3) Å	µ = 0.11 mm^−^^1^
*c* = 7.2900 (15) Å	*T* = 291 (1) K
*V* = 815.4 (3) Å^3^	Cube, colourless
*Z* = 4	0.2 × 0.2 × 0.2 mm

### Data collection

**Table:**

Nonius KappaCCD diffractometer	899 independent reflections
Radiation source: fine-focus sealed tube	536 reflections with *I* > 2σ(*I*)
Monochromator: graphite	*R*_int_ = 0.045
Detector resolution: 19 vertical, 18 horizontal pixels mm^-1^	θ_max_ = 26.4º
*T* = 291(1) K	θ_min_ = 3.9º
213 frames via ω–rotation (Δω = 1%) and two times 40 s per frame (four sets at different κ–angles) scans	*h* = −10→10
Absorption correction: none	*k* = −16→16
3104 measured reflections	*l* = −9→9

### Refinement

**Table:**

Refinement on *F*^2^	Secondary atom site location: difference Fourier map
Least-squares matrix: full	Hydrogen site location: inferred from neighbouring sites
*R*[*F*^2^ > 2σ(*F*^2^)] = 0.027	H-atom parameters constrained
*wR*(*F*^2^) = 0.057	*w* = 1/[σ^2^(*F*_o_^2^) + (0.0206*P*)^2^] where *P* = (*F*_o_^2^ + 2*F*_c_^2^)/3
*S* = 1.09	(Δ/σ)_max_ < 0.001
899 reflections	Δρ_max_ = 0.08 e Å^−^^3^
104 parameters	Δρ_min_ = −0.11 e Å^−^^3^
1 restraint	Extinction correction: SHELXL97 (Sheldrick, 2008), Fc^*^=kFc[1+0.001xFc^2^λ^3^/sin(2θ)]^-1/4^
Primary atom site location: structure-invariant direct methods	Extinction coefficient: 0.087 (6)

### Special details

**Table:**

Geometry. All e.s.d.'s (except the e.s.d. in the dihedral angle between two l.s. planes) are estimated using the full covariance matrix. The cell e.s.d.'s are taken into account individually in the estimation of e.s.d.'s in distances, angles and torsion angles; correlations between e.s.d.'s in cell parameters are only used when they are defined by crystal symmetry. An approximate (isotropic) treatment of cell e.s.d.'s is used for estimating e.s.d.'s involving l.s. planes.
Refinement. Refinement of *F*^2^ against ALL reflections. The weighted *R*-factor *wR* and goodness of fit *S* are based on *F*^2^, conventional *R*-factors *R* are based on *F*, with *F* set to zero for negative *F*^2^. The threshold expression of *F*^2^ > σ(*F*^2^) is used only for calculating *R*-factors(gt) *etc*. and is not relevant to the choice of reflections for refinement. *R*-factors based on *F*^2^ are statistically about twice as large as those based on *F*, and *R*- factors based on ALL data will be even larger.

### Fractional atomic coordinates and isotropic or equivalent isotropic displacement parameters (Å^2^)

**Table:**

	*x*	*y*	*z*	*U*_iso_*/*U*_eq_	
O1	0.14686 (19)	0.12432 (11)	0.8457 (2)	0.0633 (5)	
O2	0.1931 (2)	0.03995 (12)	0.4285 (2)	0.0784 (6)	
O3	0.4017 (2)	0.23981 (12)	0.3446 (3)	0.0923 (7)	
O4	0.4114 (2)	0.03491 (11)	0.6049 (2)	0.0634 (5)	
N1	0.19750 (19)	0.20788 (12)	0.7495 (3)	0.0536 (5)	
C1	0.2847 (3)	0.07762 (15)	0.5349 (3)	0.0520 (6)	
C3	0.2673 (2)	0.18321 (15)	0.5998 (3)	0.0463 (5)	
C5	0.3291 (3)	0.26405 (17)	0.4804 (3)	0.0557 (6)	
C7	0.0702 (3)	0.15466 (19)	1.0136 (3)	0.0749 (8)	
H7A	0.0349	0.0967	1.0796	0.112*	
H7B	−0.0205	0.1961	0.9857	0.112*	
H7C	0.1449	0.1914	1.0875	0.112*	
C8	0.4383 (4)	−0.06865 (16)	0.5537 (3)	0.0819 (9)	
H8A	0.5226	−0.0960	0.6279	0.123*	
H8D	0.4683	−0.0721	0.4268	0.123*	
H8B	0.3416	−0.1061	0.5727	0.123*	
C9	0.3008 (3)	0.37018 (15)	0.5307 (4)	0.0674 (7)	
H9A	0.3481	0.4128	0.4397	0.101*	
H9B	0.3484	0.3836	0.6480	0.101*	
H9D	0.1875	0.3826	0.5367	0.101*	

### Atomic displacement parameters (Å^2^)

**Table:**

	*U*^11^	*U*^22^	*U*^33^	*U*^12^	*U*^13^	*U*^23^
O1	0.0881 (11)	0.0498 (9)	0.0519 (9)	−0.0045 (8)	0.0208 (9)	0.0002 (9)
O2	0.0868 (13)	0.0657 (11)	0.0828 (13)	0.0028 (9)	−0.0205 (12)	−0.0202 (11)
O3	0.1322 (17)	0.0691 (12)	0.0755 (13)	−0.0101 (10)	0.0492 (14)	−0.0068 (11)
O4	0.0676 (10)	0.0551 (9)	0.0675 (10)	0.0137 (8)	−0.0078 (9)	−0.0037 (9)
N1	0.0625 (12)	0.0471 (11)	0.0510 (12)	−0.0028 (9)	0.0046 (12)	0.0004 (10)
C1	0.0601 (14)	0.0494 (13)	0.0467 (14)	−0.0018 (13)	0.0041 (14)	−0.0023 (13)
C3	0.0483 (12)	0.0491 (13)	0.0415 (12)	0.0010 (10)	−0.0001 (12)	−0.0037 (12)
C5	0.0615 (16)	0.0567 (15)	0.0490 (14)	−0.0031 (12)	0.0063 (13)	−0.0008 (13)
C7	0.0981 (19)	0.0731 (17)	0.0535 (16)	−0.0063 (16)	0.0276 (15)	−0.0040 (14)
C8	0.1075 (19)	0.0597 (17)	0.079 (2)	0.0278 (14)	0.0040 (16)	0.0008 (15)
C9	0.0773 (15)	0.0517 (14)	0.0732 (17)	−0.0057 (13)	0.0087 (13)	0.0009 (15)

### Geometric parameters (Å, °)

**Table:**

O1—N1	1.387 (2)	C7—H7A	0.9600
O1—C7	1.440 (3)	C7—H7B	0.9600
O2—C1	1.200 (3)	C7—H7C	0.9600
O3—C5	1.205 (3)	C8—H8A	0.9600
O4—C1	1.306 (3)	C8—H8D	0.9600
O4—C8	1.455 (2)	C8—H8B	0.9600
N1—C3	1.281 (3)	C9—H9A	0.9600
C1—C3	1.500 (3)	C9—H9B	0.9600
C3—C5	1.483 (3)	C9—H9D	0.9600
C5—C9	1.489 (3)		
			
N1—O1—C7	109.67 (16)	O1—C7—H7C	109.5
C1—O4—C8	116.29 (19)	H7A—C7—H7C	109.5
C3—N1—O1	111.12 (17)	H7B—C7—H7C	109.5
O2—C1—O4	125.7 (2)	O4—C8—H8A	109.5
O2—C1—C3	122.6 (2)	O4—C8—H8D	109.5
O4—C1—C3	111.7 (2)	H8A—C8—H8D	109.5
N1—C3—C5	118.01 (19)	O4—C8—H8B	109.5
N1—C3—C1	123.8 (2)	H8A—C8—H8B	109.5
C5—C3—C1	118.1 (2)	H8D—C8—H8B	109.5
O3—C5—C3	117.4 (2)	C5—C9—H9A	109.5
O3—C5—C9	122.7 (2)	C5—C9—H9B	109.5
C3—C5—C9	119.9 (2)	H9A—C9—H9B	109.5
O1—C7—H7A	109.5	C5—C9—H9D	109.5
O1—C7—H7B	109.5	H9A—C9—H9D	109.5
H7A—C7—H7B	109.5	H9B—C9—H9D	109.5

## References

[bb1] Buehler, E. (1967). *J. Org. Chem.* **32**, 261–265.

[bb2] Corrêa, I. R. Jr & Moran, P. J. S. (1999). *Tetrahedron*, **55**, 14221–14232.

[bb3] Fletcher, M. D., Hurst, T. E., Miles, T. J. & Moody, C. J. (2006). *Tetrahedron*, **62**, 5454–5463.

[bb4] François, D., Madden, A. & Murray, W. V. (2004). *Org. Lett.* **6**, 1931–1934. 10.1021/ol049640n 15176786

[bb5] Jirman, J., Lycka, A. & Ludwig, M. (1990). *Collect. Czech. Chem. Commun.* **55**, 136–146.

[bb6] Karabatsos, G. J. & Taller, R. A. (1968). *Tetrahedron*, **24**, 3347–3360.

[bb7] Levy, G. C. & Nelson, G. L. (1972). *J. Am. Chem. Soc.* **94**, 4897–4901.

[bb8] Lu, J.-Y. & Arndt, H.-D. (2007). *J. Org. Chem.* **72**, 4205–4212. 10.1021/jo0703505 17447818

[bb9] Nonius (1998). *COLLECT* Nonius BV, Delft, The Netherlands.

[bb10] Otwinowski, Z. & Minor, W. (1997). *Methods in Enzymology*, Vol. 276, *Macromolecular Crystallography*, Part A, edited by C. W. Carter Jr & R. M. Sweet, pp. 307–326. New York: Academic Press.

[bb11] Sheldrick, G. M. (2008). *Acta Cryst.* A**64**, 112–122. 10.1107/S0108767307043930 18156677

[bb12] Spek, A. L. (2003). *J. Appl. Cryst.* **36**, 7–13.

